# Omnidirectional Manipulation of Microparticles on a Platform Subjected to Circular Motion Applying Dynamic Dry Friction Control

**DOI:** 10.3390/mi13050711

**Published:** 2022-04-30

**Authors:** Sigitas Kilikevičius, Kristina Liutkauskienė, Ernestas Uldinskas, Ribal El Banna, Algimantas Fedaravičius

**Affiliations:** Department of Transport Engineering, Kaunas University of Technology, Studentų St. 56, 51424 Kaunas, Lithuania; kristina.liutkauskiene@ktu.lt (K.L.); ernestas.uldinskas@ktu.lt (E.U.); ribal.el@ktu.edu (R.E.B.); algimantas.fedaravicius@ktu.lt (A.F.)

**Keywords:** micromanipulation, microparticles, motion control, vibrations, dry friction, control, oscillating platform

## Abstract

Currently used planar manipulation methods that utilize oscillating surfaces are usually based on asymmetries of time, kinematic, wave, or power types. This paper proposes a method for omnidirectional manipulation of microparticles on a platform subjected to circular motion, where the motion of the particle is achieved and controlled through the asymmetry created by dynamic friction control. The range of angles at which microparticles can be directed, and the average velocity were considered figures of merit. To determine the intrinsic parameters of the system that define the direction and velocity of the particles, a nondimensional mathematical model of the proposed method was developed, and modeling of the manipulation process was carried out. The modeling has shown that it is possible to direct the particle omnidirectionally at any angle over the full 2*π* range by changing the phase shift between the function governing the circular motion and the dry friction control function. The shape of the trajectory and the average velocity of the particle depend mainly on the width of the dry friction control function. An experimental investigation of omnidirectional manipulation was carried out by implementing the method of dynamic dry friction control. The experiments verified that the asymmetry created by dynamic dry friction control is technically feasible and can be applied for the omnidirectional manipulation of microparticles. The experimental results were consistent with the modeling results and qualitatively confirmed the influence of the control parameters on the motion characteristics predicted by the modeling. The study enriches the classical theories of particle motion on oscillating rigid plates, and it is relevant for the industries that implement various tasks related to assembling, handling, feeding, transporting, or manipulating microparticles.

## 1. Introduction

The manipulation of microparticles is very important for many disciplines and sectors, such as micromachine technology, biotechnology, cell biology, material processing, semiconductor industries, and neuroscience [1,2,3,4,5,6,7]. Handling, transportation, and manipulation of microparticles or bulk and granular materials can be implemented by various methods and approaches that can generally be divided into two types: prehensile and non-prehensile. Prehensile methods usually involve some sort of force or form closure that is associated with grasping by microgrippers [8,9,10,11,12]. However, prehensile methods are most suited for the manipulation of individual objects, they always involve some mechanical effect on the object to be manipulated. They still struggle with precise force feedback at microscales, and the technological equipment used to perform micromanipulation tasks is usually very complex and expensive. During the processes of nonprehensile manipulation, the objects to be manipulated are subjected only to unilateral constraints. Therefore, the external mechanical effects acting on the object to be manipulated are reduced to a minimum, and the parts can be transported without damage. In addition, nonprehensile manipulation methods can typically offer lower equipment costs, larger workspaces, and shorter operational times.

Various non-prehensile manipulation methods are being used in practice and studied in the scientific literature. For example, nonprehensile manipulation operations can be performed by employing the devices that carry the objects to be moved [13], by pushing with robot end-effectors [14,15], and by controlling actuator arrays mounted under a flexible surface [16], etc. Micropositioning platforms take a significant share among nonprehensile manipulation methods and have attracted a lot of attention in recent years. Ablay [17] studied a magnetic micromanipulator with a model-free controller and a linear controller for the manipulation of microparticles in a fluid. Li et al. [18] presented a nanopositioning system composed of flexible beams mounted with magnetorheological elastomers. The properties of the beams, such as stiffness and damping, were able to be tuned under the influence of magnetic fields. Ferrara-Bello et al. [19] applied a micropositioning system actuated by three piezoelectric stacks to control the position and displacement in the three dimensions along the XYZaxis. The main disadvantage of micropositioning platforms is the limited size of their workspaces.

Microparticle manipulation implemented through vibration-assisted and acoustic techniques is widely used and investigated [20] as it is suitable for the manipulation of large numbers of microparticles without causing unwanted mechanical stress or other adverse effects such as contamination [21].

One of the acoustic methods that is suitable for microparticles is called acoustic levitation. This method exploits the acoustic radiation force in order to move microparticles that are sustained in the air [22,23]. Acoustic manipulation in microfluidic systems has recently gained significant attention in the field of biomedicine due to the potential to control individual particles, cells, or cellular clusters [24,25,26,27].

Vibration-assisted techniques utilize mechanical vibrations of the manipulation device to transport particles along a certain direction. Large workspaces and low operational times can be achieved using this approach. Asymmetry is an essential condition to achieve the motion of an object placed on a vibrating platform. It results in friction forces that are not canceled out over one cycle of vibrations. Several types of asymmetries are being employed for nonprehensile manipulation, such as time-asymmetries, kinematic asymmetries, wave asymmetries, or power asymmetries.

A time-asymmetry can be achieved through an asymmetric excitation of the platform when the forward motion takes a longer time compared to the backward motion in every cycle of the excitation [28]. This asymmetry can also be called a temporal or vibrational asymmetry. The dynamics of a body moving along a straight-line trajectory on a plate subjected to this kind of excitation were studied by Reznik et al. [28]. The dynamics of stick-slip motion under time-asymmetry were studied by Mayyas [29,30]. The time-asymmetry was created by mounting a platform on a nonlinear leaf spring that exhibits direction-dependent elasticity. Due to this peculiarity, the forward and backward accelerations of the plate are not equal when the plate is subjected to vibrational excitation.

Another type of asymmetry is kinematic asymmetry. It is implemented through an asymmetry of the vibration path or an asymmetry of the law of motion along this path. For example, this kind of asymmetry can be created when the direction of the harmonic oscillations is inclined with respect to the manipulation surface, i.e., the direction of the motion of particles to be manipulated. This asymmetry is usually applied to various vibratory conveyors and feeders [31,32,33]. Frei et al. [34] proposed a method for the manipulation of objects in individual paths by employing an array of multiple cells excited in two directions that caused a kinematic asymmetry. Vrublevskyi [35] studied the process of vibrational conveying with a kinematic asymmetry where an inclined surface was subjected to harmonic longitudinal and polyharmonic normal oscillations.

Wave asymmetries are also widely used to achieve the directional motion of particles. This kind of asymmetry is achieved by exciting traveling waves [36,37,38]. Various types of waves are used to implement this type of asymmetry. The mechanism of transport of dielectric particles on a conveyor employing a traveling electric field wave was investigated by Zouaghi et al. [39]. A wave asymmetry was employed by Kumar and DasGupta [40] to manipulate particles on a plate subjected to traveling circumferential harmonic waves.

The directional motion of an object placed on an oscillating surface can also be achieved through asymmetries classified as power asymmetries. These asymmetries can be subcategorized into types such as geometric and force asymmetries. One way to create a power asymmetry is to incline the system with respect to the horizontal plane. In this case, such an asymmetry can also be called a geometric asymmetry. Viswarupachari et al. [41] employed a geometric asymmetry along with a time-asymmetry to transport particles placed on a platform that was subjected to asymmetric vibrations. A power asymmetry can also be achieved when a constant force is applied to the object or when the resistance forces during the forward motions are not equal to the resistance forces during the backward motion. Asymmetries created in such a way can be designated as force asymmetries. Mitani et al. [42,43,44] applied an oscillating platform with a textured surface for the feeding of microparts. The motion of the microparts was achieved through a force asymmetry created by the anisotropic friction properties of the textured surface. Chen et al. proposed an oscillating trough with fin-like asperities for the transportation of particles. The fin-like asperities created a force asymmetry, which resulted in the directional motion of the particles.

Recently, a method of bidirectional vibrational transportation was demonstrated that was achieved through an asymmetry created by dynamically controlling the frictional conditions between the object being manipulated and the platform subjected to harmonic (sinusoidal) oscillations along the horizontal direction [45,46,47].

Unlike the omnidirectional manipulation systems with oscillating surfaces that are usually based on asymmetries of time, kinematic, wave, or power types, the presented work examines the case where the effective coefficient of friction is being dynamically controlled during each rotation cycle of the platform in such a way as to achieve the asymmetry of frictional conditions. This ensures the ability to manipulate various small particles on the platform in a complex trajectory. The objectives of the research are to determine the intrinsic parameters of the system that define the direction and velocity of the particles and to experimentally verify that the asymmetry created by dynamic dry friction control is technically feasible and can be applied for the omnidirectional manipulation of microparticles. The work addresses the scientific problem of manipulation at small scales, and the novelty of the research is that the motion of microparticles on a platform subjected to circular motion is achieved and controlled through the asymmetry created by dynamic friction control.

## 2. Methodology

### 2.1. Mathematical Model

**Figure 1:**
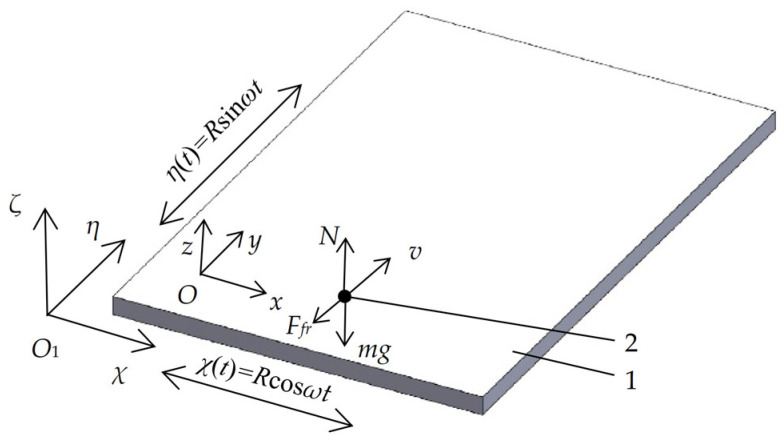
Scheme of the dynamic model of omnidirectional manipulation of particles on a platform subjected to circular motion: (1) platform; (2) particle.

Figure 1 shows a scheme of the dynamic model of omnidirectional manipulation of particles on a platform subjected to circular motion.

In this scheme, the stationary coordinate system is *O*_1_*χηζ*, and the coordinate system *Oxyz* is moving along with the rotating platform. Then, the coordinates of point ($\chi_{i},\;\eta_{i}$) on the platform in the stationary coordinate system are as follows:

(1) $$\{\begin{array}{c} \chi \left(t\right)=\chi_{i}+R\cos\omega t\\ \eta \left(t\right)=\eta_{i}+R\sin\omega t \end{array}$$

where *t* is the time, *R* is the radius of the circular motion, and *ω* is the angular frequency of the circular motion.

In the *0xy* coordinate system, the relative motion of the particle on the platform subjected to circular motion is described by the following differential equations:

(2) $$\{\begin{array}{c} \ddot{x}+\frac{g\mu \left(t\right)\dot{x}}{\sqrt{{\dot{x}}^{2}+{\dot{y}}^{2}}}=R\omega^{2}\cos\omega t\\ \ddot{y}+\frac{g\mu \left(t\right)\dot{y}}{\sqrt{{\dot{x}}^{2}+{\dot{y}}^{2}}}=R\omega^{2}\sin\omega t \end{array}$$

where ${\dot{x}}^{2}+{\dot{y}}^{2}\neq 0$, μ(t) is the coefficient of dry friction, which is being controlled with respect to the period of the circular motion of the platform in order to achieve the asymmetry of frictional conditions.

Nondimensionalization was applied to determine the intrinsic parameters of the system that define the direction and velocity of particles. In order to nondimensionalize Equation (2), the following nondimensional parameters were introduced:

(3) $$\xi^{\prime }=\frac{\dot{x}}{R\omega };\;\psi^{\prime }=\frac{\dot{y}}{R\omega };\gamma =\frac{g}{R\omega^{2}};\tau =\omega t$$

Differentiation with respect to nondimensional time *τ* is denoted using the Lagrange notation. In this case, the nondimensionalized equations of motion can be written as follows:

(4) $$\{\begin{array}{c} \xi \prime \prime +\frac{\mu \left(\tau \right)\gamma \xi^{\prime }}{\sqrt{{\xi^{\prime }}^{2}+{\psi^{\prime }}^{2}}}=\cos\tau \\ \psi \prime \prime +\frac{\mu \left(\tau \right)\gamma \psi^{\prime }}{\sqrt{{\xi^{\prime }}^{2}+{\psi^{\prime }}^{2}}}=\sin\tau \end{array}$$

where ${\xi^{\prime }}^{2}+{\psi^{\prime }}^{2}\neq 0$, ξ and ψ are the horizontal and vertical components of the nondimensional displacement, respectively.

**Figure 2:**
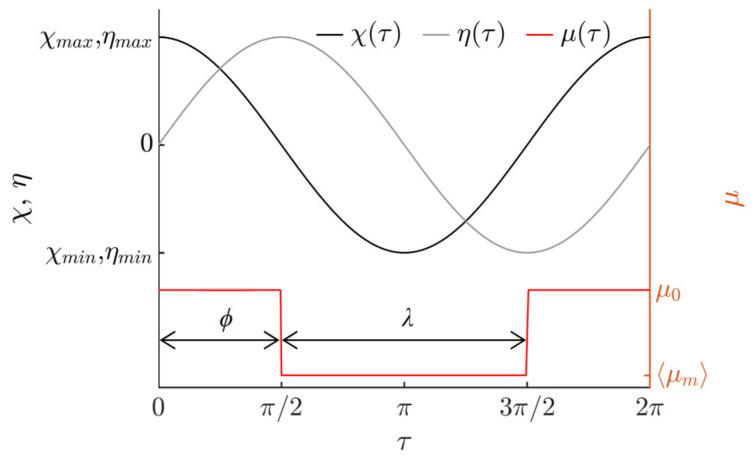
Principle of dynamic dry friction control.

The dry friction coefficient *μ*(*τ*) is controlled with respect to the circular motion of the platform by the following function:

(5) $$\mu \left(\tau \right)=\{\begin{array}{l} \langle \mu_{m}\rangle ,\;\mathrm{when}\;2\pi n+\phi <\tau <2\pi n+\phi +\lambda ,\\ \mu_{0},\;\;\mathrm{otherwise}, \end{array}$$

where *n* = (0, 1, 2, …), *µ*_0_ is the nominal dry friction coefficient between the particle and the platform’s surface, ⟨*µ_m_*⟩ is the dynamically modified time-averaged effective dry friction coefficient between the particle and the platform’s surface, *ϕ* is the phase shift between the function governing the circular motion and the dry friction control function, and λ is the width of the dry friction control function. The principle of dynamic dry friction control is shown in Figure 2.

The average nondimensional velocity of the particle can be found by the following:

(6) $$\langle \vartheta \rangle =\frac{1}{2\pi }\overset{2\pi }{\underset{0}{\int }}\sqrt{{\xi^{\prime }}^{2}+{\psi^{\prime }}^{2}}d\tau$$

Equation (4) can also be expressed in the polar coordinate system by defining the horizontal and vertical components of the nondimensional velocity as follows:

(7) $$\xi^{\prime }=\rho \cos\theta$$

(8) ψ′=ρsinθ

Then, Equation (4) in the polar coordinate system can be written as follows:

(9) $$\{\begin{array}{c} \rho^{\prime }+\mu \left(\tau \right)\gamma =\cos\left(\tau -\theta \right)\\ \rho \theta^{\prime }=\sin\left(\tau -\theta \right) \end{array}$$

where ρ≠0, *ρ* and *θ* are the magnitude and phase of the angular velocity vector, respectively.

### 2.2. Methodology of Experimental Investigation

**Figure 3:**
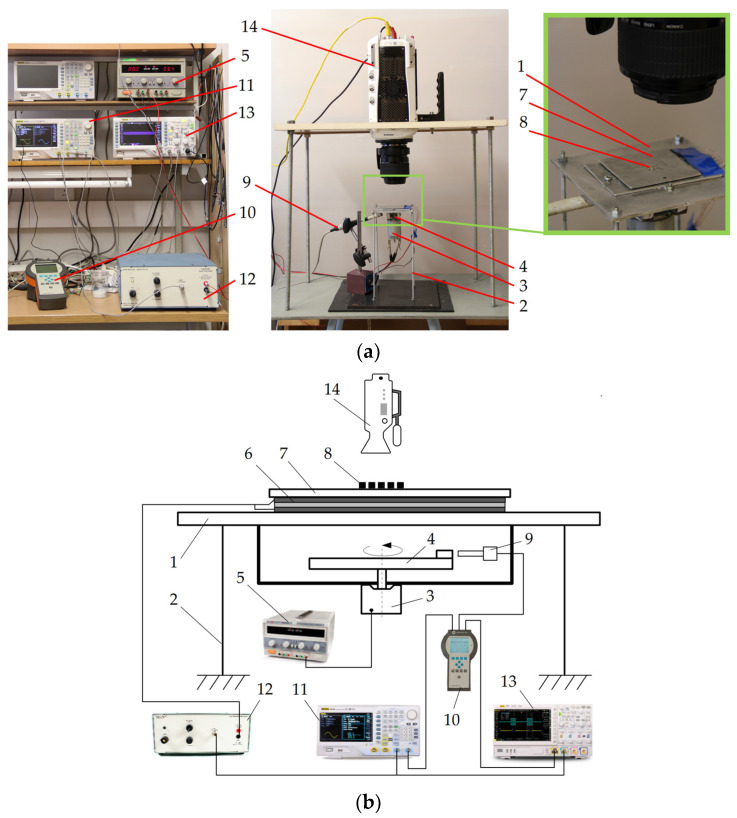
Experimental setup for omnidirectional manipulation applying dynamic dry friction control: (**a**) General view; (**b**) Scheme where the following components are shown: (1) platform; (2) elastic rods; (3) piezoelectric actuator; (4) eccentric mechanism; (5) direct current power supply; (6) piezoelectric actuators; (7) manipulation plate; (8) microparticles; (9) optical reference sensor; (10) vibration analyzer; (11) arbitrary waveform generator; (12) piezo linear amplifier; (13) digital oscilloscope; (14) high-speed camera.

Figure 3a shows a general view of the experimental setup built for omnidirectional manipulation of microparticles on a platform subjected to circular motion applying dynamic dry friction control. Figure 3a displays a schematic of the experimental setup that was used for the investigation. The setup consists of a platform (1) sustained by four elastic rods (2) (Figure 3a). The platform is subjected to circular motion by an electric motor (3) with an eccentric mechanism (4). A direct current power supply (HY3002-2, Mastech, Shenzhen, China) (5) supplies power to the electric motor. Four rectangular piezoelectric actuators (6) are mounted on the platform to excite a manipulation plate (7) in the vertical direction. The upper surface of the manipulation plate is polished to an average surface roughness of about 0.44 mm. The manipulation of microparticles (8) takes place on this surface. The phase of the circular motion of the platform is monitored by an optical reference sensor (P–95, Brüel & Kjær, Nærum, Denmark) (9). The sensor signal is processed by a vibration analyzer (Vibrotest 60, Brüel & Kjær, Nærum, Denmark) (10).

The signal for dry friction control is composed of high-frequency pulses in burst mode that are generated by an arbitrary waveform generator (DG4202, RIGOL, Beijing, China) (11) and amplified by a piezo linear amplifier (EPA-104, Piezo Systems Inc., Cambridge, MA, USA) (12) and fed to the piezoelectric actuators. This signal is synchronized with respect to the phase of the circular excitation. Based on the principle of dry friction control shown in Figure 2, the piezoelectric actuators are being excited by a frequency of 2094 Hz for a fraction equal to *λ* and shifted by *ϕ* in each period of the circular motion of the manipulation plate. It has been demonstrated numerous times before that high-frequency vibrations introduced between sliding objects cause a reduction in the effective dry friction force between these objects as a result of the dynamic processes that occur in the contact region [48,49,50]. Therefore, in this fraction of the period when the manipulation plate is subjected to high-frequency vibrations by the piezoelectric actuators, the effective friction force between the manipulation plate’s surface and the particle is reduced. In this way, the dry friction force can be controlled in a predefined manner with respect to the period of the circular motion of the manipulation plate. The dry friction control signal and the optical reference sensor readout are monitored and displayed by a digital oscilloscope (DS1054, RIGOL, Beijing, China) (13).

A high-speed camera (Phantom v711, 1280 × 800 CMOS sensor, 1 Mpx, 20 µm pixel size, Vision Research, Wayne, NJ, USA) (14) equipped with a macro lens (MP-E 65 mm f/2.8 1–5x, Canon Inc., Ōta, Tokyo, Japan) was used to record the motion of the particles. A video processing program based on the normalized cross-correlation approach was developed using MATLAB to digitize and analyze the motion of the particles.

## 3. Results

### 3.1. Modeling Results

Numerical modeling of omnidirectional manipulation on a platform subjected to a circular motion under dynamic friction control was carried out. For this purpose, software was developed in the MATLAB programming language (MathWorks, Natick, MA, USA). The equations were solved using the ode45 solver based on the Runge–Kutta (4, 5) formula and the Dormand–Prince pair.

**Figure 4:**
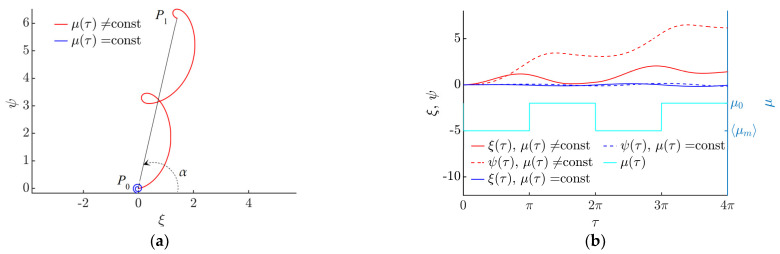
Nondimensional displacement of the particle when *γ* = 4.9, *µ*_0_ = 0.2, ⟨*µ_m_*⟩/*µ*_0_ = 0.25, *λ* = *π*, *ϕ* = 0: (**a**) motion trajectories of the particle and the angle of displacement *α*. (**b**) horizontal *ξ* and vertical *ψ* components of the nondimensional displacement vs. the nondimensional time and the variation of the effective dry friction coefficient.

In this study, the straight line between the starting and endpoints (Figure 4a, where *P*_0_ is the starting point and *P*_1_ is the endpoint) was assumed to be the distance covered by the particle. The angle between the distance covered by the particle and the horizontal axis was assumed to be the displacement angle *α*.

The modeling has revealed that the motion of a particle can be achieved through the asymmetry created by dynamic friction control (Figure 4a). Figure 4a shows the trajectories of the particles after two cycles of the circular motion of the platform. The blue trajectory represents the motion of the particle on the platform when the dry friction between the part and the platform is not being controlled. In this case, the particle is moving around the position of equilibrium (Figure 4a when *µ* = const), as shown in Figure 4b, where the horizontal *ξ* and vertical *ψ* components of the nondimensional displacement oscillate around a constant value (Figure 4b when *µ* = const). The red trajectory represents the motion of the particle when the dry friction coefficient between the part and the platform is being controlled, i.e., it is being periodically modified for some fraction of the period. In this case, the system’s symmetry is eliminated, and the particle is constantly moving in a certain direction (Figure 4a when *µ* ≠ const). Figure 4b shows how the variation of the effective friction coefficient influences the horizontal *ξ* and vertical *ψ* components of the nondimensional displacement. An increase in the magnitudes of *ξ* and *ψ* is starting to take place periodically in the intervals of *τ* where the friction coefficient is dynamically modified to be equal to ⟨*µ_m_*⟩.

**Figure 5:**
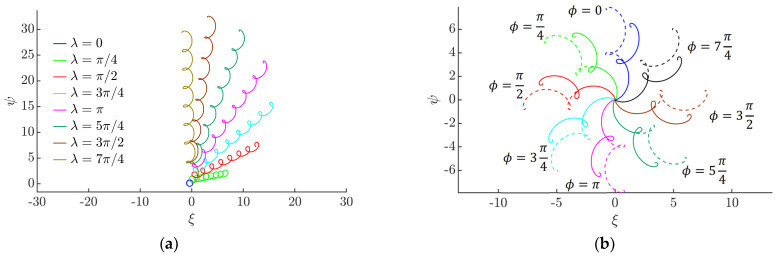
Trajectories of the particle: (**a**) under different values of *λ* after 8 cycles of the circular motion of the platform when *ϕ =* 0, *µ*_0_ = 0.2, ⟨*µ_m_*⟩/*µ*_0_ = 0.25, *γ* = 4; (**b**) under different values of the phase shift *ϕ* after two cycles when *µ*_0_ = 0.2, ⟨*µ_m_*⟩/*µ*_0_ = 0.25, *γ* = 4.9, *λ = π* (solid line), *λ =* 13*π*/9 (dashed line).

The shape of the trajectory of the particle depends mainly on *λ* (Figure 5a). In a symmetric system (when *λ* = 0), the particle can travel some distance from the starting point in an undulating or spiral trajectory, but then it starts to circle around the point of equilibrium [51,52]. As a result of the asymmetry created by dynamic dry friction control, the particle can be moved in a preferred direction. Figure 5a shows that the shape of the trajectory becomes less circular and undulating, and the particle travels a greater distance from the starting point at higher values of *λ* since an increase in *λ* results in a greater asymmetry of the system. The displacement angle depends mainly on the phase shift *ϕ* (Figure 5b).

**Figure 6:**
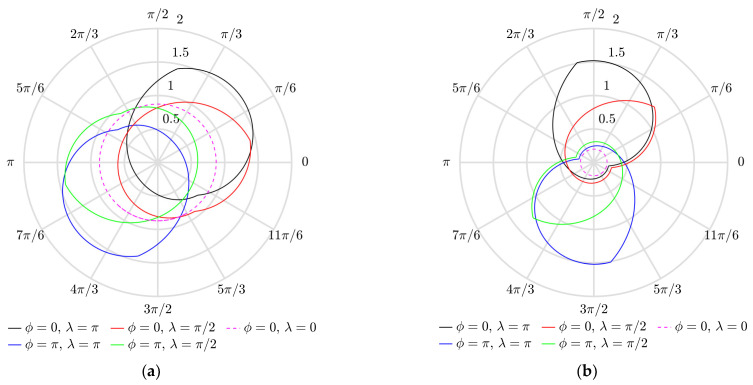
Variation of the angular velocity vector during the period of the eighth cycle of circular motion in polar coordinates when ⟨*µ_m_*⟩/*µ*_0_ = 0.25, *γ* = 4.9: (**a**) *µ*_0_ = 0.1; (**b**) *µ*_0_ = 0.2.

Figure 6 shows how the vector of the angular velocity of the particle varies during a stable period of the circular motion of the platform. In a symmetric system (when *λ* = 0), the magnitude of the vector of the angular velocity is constant (the polar plots are represented by the dashed circles in magenta). This indicates that the particle is moving around the position of equilibrium; therefore, it is not gaining displacement. The asymmetry created by dynamic dry friction control results in the asymmetric shapes of the polar plots that indicate that the particle is gaining displacement. An increase in *λ* results in an increase in the magnitude of the angular velocity vector *ρ*. The phase shift *ϕ* defines the phase of the vector of angular velocity *θ* at which the maximum value of *ρ* is reached.

**Figure 7:**
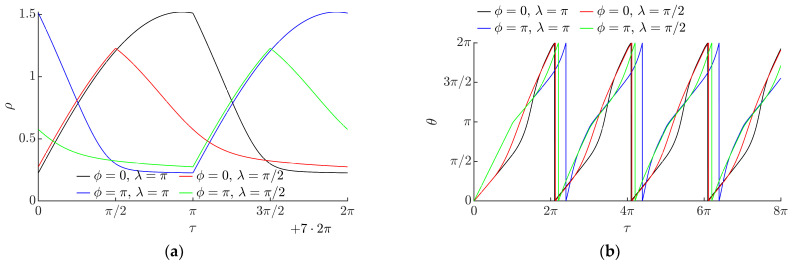
Angular velocity vector vs. the nondimensional time when *µ*_0_ = 0.2, ⟨*µ_m_*⟩/*µ*_0_ = 0.25, *γ* = 4.9: (**a**) magnitude *ρ* during the period of the 8th cycle; (**b**) phase *θ* during the first four cycles.

The variation of the angular velocity vector during a stable period of the circular motion is shown in Figure 7a. The magnitude of the angular velocity vector *ρ* oscillates over some positive value under the influence of dynamic dry friction control. The amplitude of these oscillations depends on *λ*. These oscillations indicate that the particle is gaining displacement. An increase in *λ* results in a higher value of this amplitude. Figure 7b shows how the phase of the vector of angular velocity varies over nondimensional time.

The influence of the intrinsic system parameters (*λ*, *ϕ*, *µ*_0_, *γ*, ⟨*µ_m_*⟩/*µ*_0_) on the nondimensional average velocity of the particle 〈*ϑ*〉 was determined. The nondimensional average velocity 〈*ϑ*〉 was found by dividing the nondimensional displacement gained by the particle after 17 cycles by the nondimensional time of travel.

**Figure 8:**
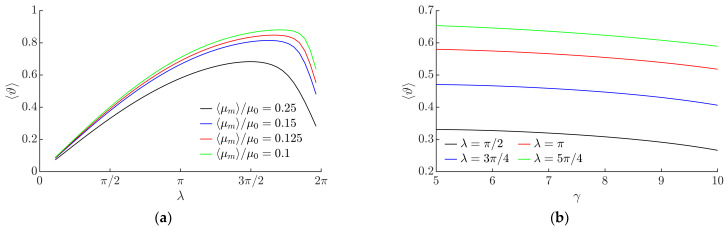

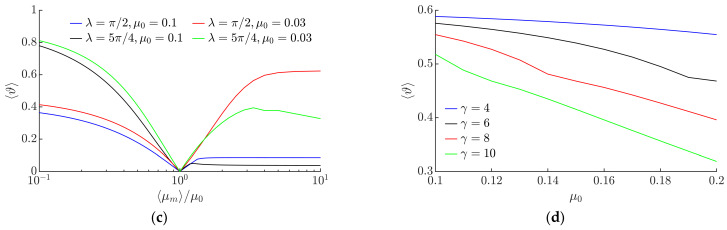
Average nondimensional velocity depending on the following: (**a**) *λ* when *µ*_0_ = 0.1, *γ* = 5, *ϕ* = 0; (**b**) *γ* when *µ*_0_ = 0.1, *ϕ* = 0, ⟨*µ_m_*⟩/*µ*_0_ = 0.25; (**c**) ⟨*µ_m_*⟩/*µ*_0_ when *γ* = 9, *ϕ* = *π*/2; (**d**) *µ*_0_ when ⟨*µ_m_*⟩/*µ*_0_ = 0.25, *ϕ* = 0, *λ = π*/2.

In a range of *λ* up to approximately 3*π*/2, the average nondimensional velocity 〈*ϑ*〉 increases when *λ* increases due to the increasing asymmetry of frictional conditions (Figure 8a). When the maximum of 〈*ϑ*〉 is reached, a further increase in λ results in a decrease in the average nondimensional velocity 〈*ϑ*〉 due to the decreasing asymmetry of fictional conditions in this range of such high values of *λ*.

Figure 8b shows the influence of *γ* on 〈*ϑ*〉. An increase in *γ* results in a slight decrease in the average nondimensional velocity 〈*ϑ*〉.

The ratio ⟨*µ_m_*⟩/*µ*_0_ represents how much the dry friction coefficient is dynamically modified with respect to the nominal dry friction coefficient. The modeling showed that this ratio has a significant influence on the average nondimensional velocity 〈*ϑ*〉 (Figure 8c). As ⟨*µ_m_*⟩/*µ*_0_ approaches 1, the average nondimensional velocity 〈*ϑ*〉 decreases until it becomes equal to 0 at ⟨*µ_m_*⟩/*µ*_0_ = 1. This is due to the fact that ⟨*µ_m_*⟩/*µ*_0_ values further from 1 result in a greater asymmetry of the system, and the system is in a symmetric state when ⟨*µ_m_*⟩/*µ*_0_ = 1. When ⟨*µ_m_*⟩/*µ*_0_ is less than 1, the friction is being periodically reduced, and when it is higher than 1, it is being periodically increased. These results show that the dry friction force can be periodically increased or decreased for some fraction of the period of the circular motion in order to achieve the asymmetry of the system by dynamic dry friction control. The red curve in Figure 8c shows that the control approach implemented by periodically decreasing the dry friction force (⟨*µ_m_*⟩/*µ*_0_ > 1) is more efficient when the nominal coefficient *µ*_0_ and *λ* are very low.

Figure 8d shows the influence of the nominal dry friction coefficient *µ*_0_ on the average nondimensional velocity at a value of ⟨*µ_m_*⟩/*µ*_0_ that is lower than 1. In this case, when *µ*_0_ increases, the average nondimensional velocity tends to decrease. The decrease is more pronounced at higher values of *γ* (Figure 8d).

The modeling has shown that the average nondimensional velocity 〈*ϑ*〉 does not depend on the phase shift *ϕ* between the function governing the circular motion and the dry friction control function.

**Figure 9:**
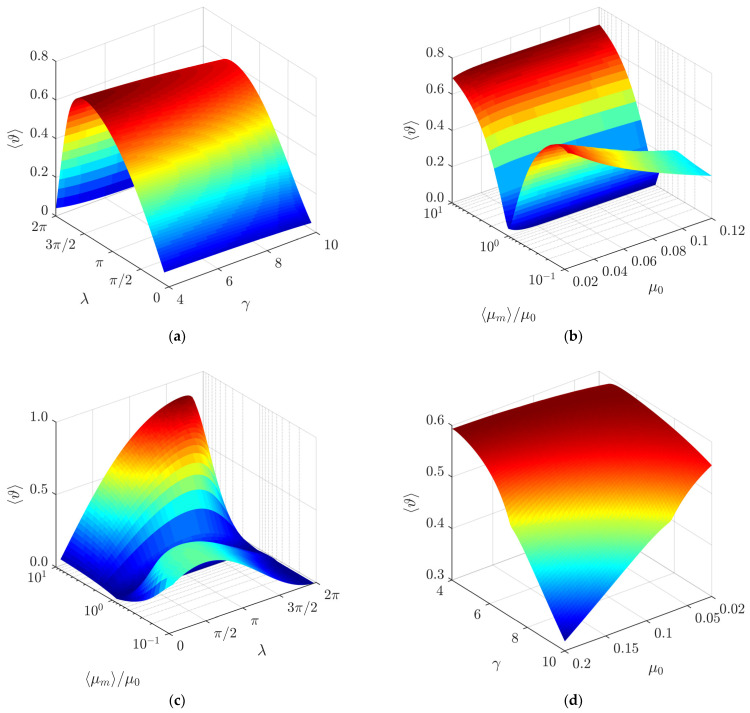
Average nondimensional velocity depending on the following: (**a**) *λ* and *γ* when *µ*_0_ = 0.1, ⟨*µ_m_*⟩/*µ*_0_ = 0.25, *ϕ* = 0; (**b**) ⟨*µ_m_*⟩/*µ*_0_ and *µ*_0_ when *γ* = 5, *ϕ* = 0, *λ = π*; (**c**) ⟨*µ_m_*⟩/*µ*_0_ and *λ* when *µ*_0_ = 0.1, *γ* = 5, *ϕ* = 0; (**d**) *γ* and *µ*_0_ when ⟨*µ_m_*⟩/*µ*_0_ = 4, *ϕ* = 0, *λ = π*.

Figure 9a shows a three-dimensional diagram of the average nondimensional velocity 〈*ϑ*〉 as a function of *λ* and *γ*. Under the analyzed conditions, the average nondimensional velocity reaches its maximum value when *λ* is near 3π/2.

The combined influence of the ratio ⟨*µ_m_*⟩/*µ*_0_ and the nominal dry friction coefficient *µ*_0_ on the average nondimensional velocity is shown in Figure 9b. In the case where the friction coefficient is being periodically decreased (⟨*µ_m_*⟩/*µ*_0_ < 1), the average nondimensional velocity mainly depends on the ratio ⟨*µ_m_*⟩/*µ*_0_, and it is less sensitive to the nominal dry friction coefficient *µ*_0_. In the case where the friction coefficient is being periodically increased (⟨*µ_m_*⟩/*µ*_0_ > 1), both *µ*_0_ and ⟨*µ_m_*⟩/*µ*_0_ have a noticeable influence on the average nondimensional velocity. Higher values are observed at lower values of the nominal dry friction coefficient *µ*_0_.

Figure 9c shows a three-dimensional diagram of the average nondimensional velocity 〈*ϑ*〉 as a function of ⟨*µ_m_*⟩/*µ*_0_ and *λ*. In the case where the friction coefficient is being periodically decreased (⟨*µ_m_*⟩/*µ*_0_ < 1), the maximum values of 〈*ϑ*〉 are obtained in a range of *λ* from 3π/2 to 7π/4 approximately. The value of *λ*, at which the maximum of 〈*ϑ*〉 is reached, depends on ⟨*µ_m_*⟩/*µ*_0_. Under values of ⟨*µ_m_*⟩/*µ*_0_ that are further from 1, the maximum of 〈*ϑ*〉 is reached at higher values of *λ*. In the case where the friction coefficient is being periodically increased (⟨*µ_m_*⟩/*µ*_0_ > 1), the asymmetry of frictional conditions is shifted with respect to the function governing the circular motion differently compared to the case where ⟨*µ_m_*⟩/*µ*_0_ < 1. Therefore, the maximum values of 〈*ϑ*〉 are obtained in a range of *λ* from approximately *π*/2 to 3*π*/4. In this case, under values of ⟨*µ_m_*⟩/*µ*_0_ that are further from 1, the maximum of 〈*ϑ*〉 is reached at lower values of *λ*.

Figure 9d shows a three-dimensional diagram of the average nondimensional velocity 〈*ϑ*〉 as a function of *γ* and *µ*_0_. A decrease in 〈*ϑ*〉 is observed with an increase in both *γ* and *µ*_0_.

**Figure 10:**
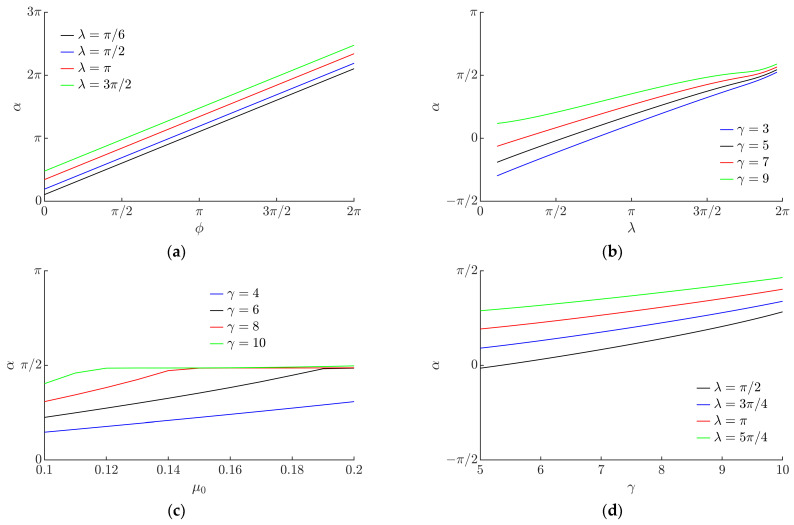
Displacement angle α depending on: (**a**) *ϕ* when *µ*_0_ = 0.1, *γ* = 8.8, ⟨*µ_m_*⟩/*µ*_0_ = 0.25; (**b**) *λ* when *µ*_0_ = 0.1, *ϕ* = 0, ⟨*µ_m_*⟩/*µ*_0_ = 0.25; (**c**) *µ*_0_ when ⟨*µ_m_*⟩/*µ*_0_ = 0.25, *ϕ* = 0, *λ = π*/2; (**d**) *γ* when *µ*_0_ = 0.1, *ϕ* = 0, ⟨*µ_m_*⟩/*µ*_0_ = 0.25.

The modeling has shown that the displacement angle α linearly depends on the phase shift *ϕ* (Figure 10a). This implies that it is possible to direct the particle omnidirectionally at any angle by changing *ϕ*.

Figure 10b shows the influence of *λ* on the displacement angle α. An increase in *λ* results in an increase in α to some extent.

An increase in the nominal dry friction coefficient *µ*_0_ has a small effect on the displacement angle α (Figure 10c). As the nominal dry friction coefficient *µ*_0_ increases, the displacement angle slightly increases when *γ* values are low.

The influence of the parameter *γ* on the displacement angle *a* is presented in Figure 10d. The modeling results demonstrate that when *γ* increases, the displacement angle slightly increases as well.

**Figure 11:**
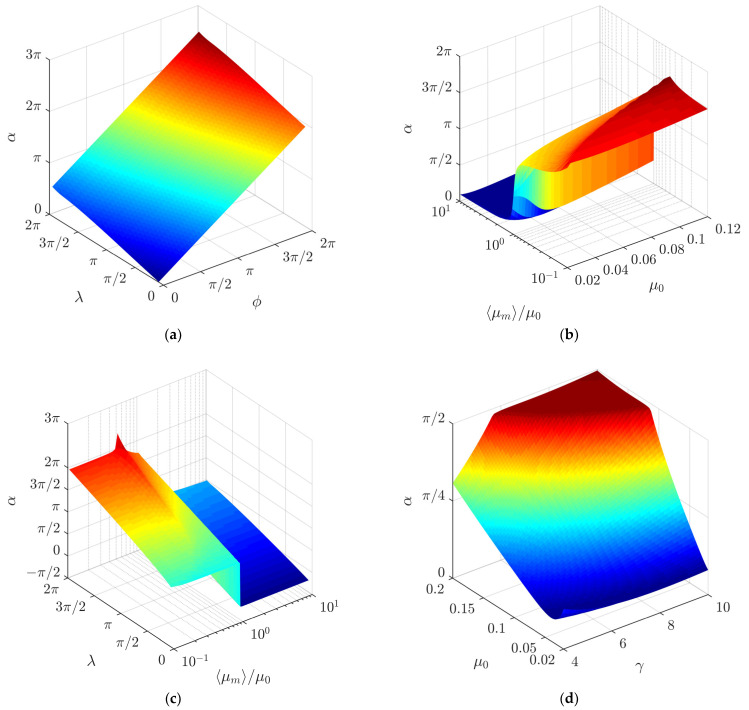
Displacement angle α depending on: (**a**) *λ* and *ϕ* when *µ*_0_ = 0.1, *γ* = 8, ⟨*µ_m_*⟩/*µ*_0_ = 0.25; (**b**) ⟨*µ_m_*⟩/*µ*_0_ and *µ*_0_ when *γ* = 5, *λ = π*, *ϕ* = 0; (**c**) *λ* and ⟨*µ_m_*⟩/*µ*_0_ when *µ*_0_ = 0.1, *γ* = 5, *ϕ* = 0; (**d**) *µ*_0_ and *γ* when ⟨*µ_m_*⟩/*µ*_0_ = 0.25, *ϕ* = 0, *λ = π*.

Figure 11a shows a three-dimensional diagram of the displacement angle *α* as a function of *λ* and *γ*. An increase in both *λ* and *γ* results in an increase in *α*.

Figure 11b shows a three-dimensional diagram of the displacement angle *α* as a function of the ratio ⟨*µ_m_*⟩/*µ*_0_ and the nominal dry friction coefficient *µ*_0_. In the case where the friction coefficient is being periodically decreased (⟨*µ_m_*⟩/*µ*_0_ < 1), the ratio ⟨*µ_m_*⟩/*µ*_0_ does not have a significant influence on the displacement angle *α*. However, an increase in *µ*_0_ results in a slight increase in *α* in this case. In the case where the friction coefficient is being periodically increased (⟨*µ_m_*⟩/*µ*_0_ > 1), both ⟨*µ_m_*⟩/*µ*_0_ and *µ*_0_ have some influence on *α*. When ⟨*µ_m_*⟩/*µ*_0_ increases, the displacement angle *α* slightly increases until it reaches a critical value and then starts to decrease slightly again. The value of ⟨*µ_m_*⟩/*µ*_0_, at which this critical value is reached, depends on *µ*_0_. Under higher values of *µ*_0_, the critical value is reached at lower values of ⟨*µ_m_*⟩/*µ*_0_. The displacement angle α in the case where ⟨*µ_m_*⟩/*µ*_0_ > 1 is shifted compared to the case when ⟨*µ_m_*⟩/*µ*_0_ < 1. This can be explained by the fact that the asymmetry of frictional conditions is shifted differently with respect to the function governing the circular motion in these two cases.

A similar shift is observed in a three-dimensional diagram of the displacement angle as a function of *λ* and ⟨*µ_m_*⟩/*µ*_0_ (Figure 11c). Figure 11c shows a similar influence of ⟨*µ_m_*⟩/*µ*_0_ on *α,* as it was discussed previously. It also shows that *λ* has a more significant influence on *α*. The displacement angle *α* increases with an increase in *λ*.

Figure 11d shows a three-dimensional diagram of the displacement angle *α* as a function of *µ*_0_ and *γ*. At higher values of both *µ*_0_ and *γ*, *α* does not change much under the influence of these parameters. A further decrease in *µ*_0_ results in a decrease in *α* as well, with an exception at very low *µ*_0_ and *γ* values.

### 3.2. Experimental Results

The proposed method of omnidirectional manipulation was experimentally tested with multilayer ceramic capacitors (MLCC) as such particles are widely used in microelectronics [42,43,44,53,54]. The developed setup was tested using 0402- and 0603-type MLCCs (Mouser Electronics, Mansfield, MA, USA).

The experiments verified that the asymmetry created by dynamic dry friction control is technically feasible and can be applied for the omnidirectional manipulation of microparticles. In the experiments, the particles were moving on the manipulation surface, and the characteristics of the motion depended on the friction control parameters *ϕ* and *λ*.

In the experiments, the average velocity of the particle ⟨*v*⟩ was calculated by dividing the distance between the starting and end points of the displacement by the time of travel.

**Figure 12:**
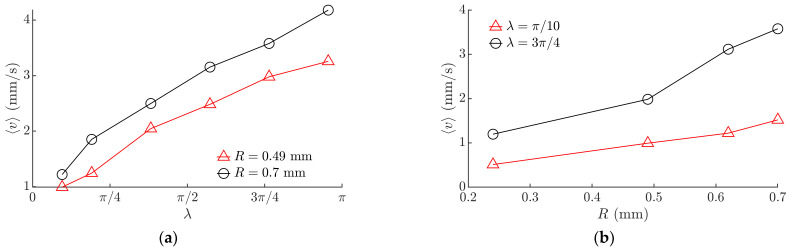
Experimental results of the average velocity depending on the following: (**a**) *λ* when *ω* = 62.83 rad/s, *ϕ* = 0; (**b**) the radius of the circular motion of the platform *R* when *ω* = 62.83 rad/s, *ϕ* = 0.

Figure 12 shows the experimental results for the average velocity of a 0603-type MLCC. An increase in λ resulted in an increase in the average velocity ⟨*v*⟩ (Figure 12a). This trend was consistent with the trend obtained by the modeling (Figure 8a). An increase in the average velocity ⟨*v*⟩ was observed with an increase in the radius of the circular motion of the platform (Figure 12b).

**Figure 13:**
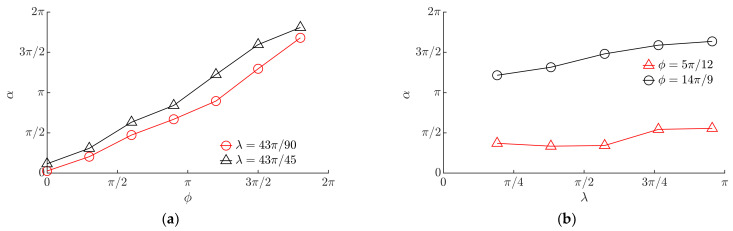
Experimental results of α depending on the following: (**a**) *ϕ* when *R* = 0.49 mm, *ω* = 62.83 rad/s; (**b**) *λ* when *R* = 0.49 mm, *ω* = 62.83 rad/s.

The experiments showed that the displacement angle mainly depends on the phase shift *ϕ*. An increase in *ϕ* resulted in a proportional increase in the displacement angle α (Figure 13a). This finding was consistent with the relationship obtained by the modeling (Figure 10a). A slight increase in the displacement angle was observed with an increase in *λ* (Figure 13b). This trend was consistent with the trend obtained by the modeling (Figure 8b).

**Figure 14:**
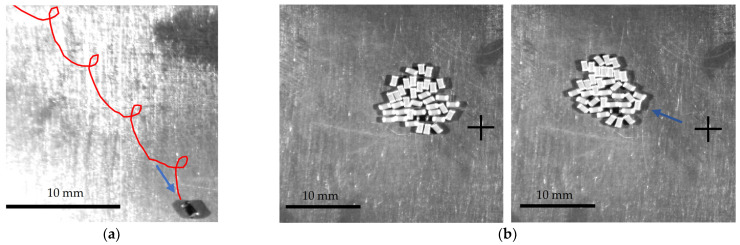
Images captured during the experiments: (**a**) captured trajectory of a single 0603-type MLCC when *R* = 0.49 mm, *ω* = 62.83 rad/s, *ϕ* = 9*π*/5, *λ =* 43*π*/90; (**b**) two frames separated by a time interval of 0.792 s that were captured during the manipulation of multiple 0603-type MLCC when *R* = 0.49 mm, *ω* = 62.83 rad/s, *ϕ* = 9*π*/10, *λ =* 43*π*/45.

Figure 14a shows an image captured during the manipulation of a single 0603-type MLCC. In this image, the trajectory of motion is shown by the red curve. The captured trajectory is very similar to the trajectories obtained by the modeling (Figure 5b).

The experiments have shown that the proposed method can be applied for the manipulation of an individual particle or multiple particles. Figure 14b shows two frames separated by a time interval of 0.792 s that were captured during the manipulation of multiple 0603-type MLCC. The motion of a single 0603-type MLCC on the manipulation plate is demonstrated in Supplementary Material Video S1. The motion of multiple MLCCs on the manipulation plate is demonstrated in Supplementary Material Video S2.

## 4. Conclusions

A method for omnidirectional manipulation of microparticles on a platform subjected to circular motion is proposed, which is achieved by applying dynamic dry friction control.

In order to determine the intrinsic parameters of the system that define the direction and velocity of particles, a nondimensional mathematical model of the proposed method was developed. Modeling of the manipulation process applying dynamic dry friction control was carried out. The modeling has revealed that the motion of a particle can be achieved and controlled through the asymmetry created by dynamic friction control. An asymmetry necessary to achieve the particle’s motion can be created when the effective friction coefficient is either periodically increased or decreased for some fraction of the period of the circular motion. The modeling has shown that it is possible to direct the particle omnidirectionally at any angle by changing the phase shift *ϕ* between the function governing the circular motion and the dry friction control function. The average nondimensional velocity does not depend on the phase shift. The shape of the trajectory of the particle depends mainly on *λ*. The shape of the trajectory becomes less circular and undulating at higher values of *λ* since an increase in *λ* results in a greater asymmetry of the system. Due to this, the velocity of the particle can be controlled through this parameter *λ*. Furthermore, an increase in λ results in an increase in the displacement angle as well. The ratio between the dynamically modified dry friction coefficient and the nominal dry friction coefficient ⟨*µ_m_*⟩/*µ*_0_ determines the size of the asymmetry. Therefore, values of ⟨*µ_m_*⟩/*µ*_0_ that are further from one result in a higher velocity. The modeling has also shown that an increase in the parameter *γ* results in a slight decrease in both the average velocity and the displacement angle. When the nominal dry friction coefficient increases, the average velocity tends to decrease.

An experimental investigation of the omnidirectional manipulation of microparticles on a horizontal platform subjected to the circular motion was carried out by implementing the method of dynamic dry friction control. The experiments verified that the asymmetry created by dynamic dry friction control is technically feasible and can be applied for the omnidirectional manipulation of microparticles. In the experiments, the particles were moving on the manipulation plate, and the characteristics of motion depended on the friction control parameters. The experimental results were consistent with the modeling results and showed that it is possible to direct the particle omnidirectionally at any angle by changing the phase shift *ϕ*. The experiments also showed that the velocity of the particle can be controlled by changing *λ*. The proposed method can be applied to the manipulation of an individual particle or multiple particles. The experiments qualitatively confirmed the influence of the control parameters on the motion characteristics predicted in the modeling, since the trends of the results observed in the experiments were in agreement with the trends of the modeling results.

Potential practical applications of the proposed method can be lab-on-a-chip applications, microassembly lines, feeders, and distributors of microparticles, handling and transportation systems of bulk materials, or other systems for manipulation of delicate components. Due to its relatively simple technical implementation, the proposed method can replace other more expensive and complex devices such as microgrippers.

The study enriches the classical theories of particle motion on oscillating rigid plates, and it is relevant for the industries that implement various tasks related to assembling, handling, feeding, transporting, or manipulating microparticles.

## Supplementary Materials

The following supporting information can be downloaded at: https://www.mdpi.com/article/10.3390/mi13050711/s1, Video S1: motion of a single particle; Video S2: motion of multiple particles on the platform subjected to circular motion applying dynamic dry friction control.
